# Left-sided perforated appendicitis in a 22-year-female with intestinal malrotation: A case report from a resource-limited setting

**DOI:** 10.1016/j.ijscr.2025.112123

**Published:** 2025-10-30

**Authors:** Abdulrahman Mohammed Abdulrahman Abouh, Ahmed Idris Abdelrahman Idris, Arwa Mohammed Abdallah Gomaa, Hussain Gadelkarim Ahmed

**Affiliations:** a Department of Surgery, El Obeid Teaching Hospital, El Obeid, North Kordofan, Sudan; b Prof Medical Research Consultancy Center, El Obeid, North Kordofan, Sudan; c Department of Histopathology and Cytology, Faculty of Medical Laboratory Sciences, University of Khartoum, Sudan

**Keywords:** Left-sided appendicitis, Malrotation, Low-resource setting, Perforated appendix, Case report

## Abstract

**Introduction and importance:**

A left-sided appendicitis is rare and usually caused by congenital anomalies such as intestinal malrotation or situs inversus. Diagnosis can be particularly challenging in low-resource settings where imaging such as CT is unavailable.

**Presentation of case:**

A 22-year-old female presented with periumbilical pain that migrated to left iliac fossa area over five days. Due to the lack of CT imaging, clinical evaluation led to a diagnosis of acute abdomen. Emergency laparotomy revealed a perforated appendix located in the left lower quadrant due to intestinal malrotation. Appendectomy was performed, and the patient recovered uneventfully. Her last menstrual period was one week before admission, and gynecologic causes were considered in the differential diagnosis but excluded intraoperatively.

**Clinical discussion:**

As a result of this case, appendicitis should be considered in the differential diagnosis of left-sided abdominal pain. The intraoperative recognition of malrotation emphasizes the importance of surgical awareness of anatomical variations, particularly when preoperative imaging is lacking.

**Conclusion:**

The diagnosis of left-sided perforated appendicitis is rare, but should be considered in patients with atypical abdominal pain. In resource-limited environments, clinical judgment and timely surgical exploration are essential.

## Highlights

• Left-sided appendicitis is rare and often caused by malrotation or situs inversus.
• Diagnosis can be difficult in low-resource settings without CT imaging.
• Timely surgical exploration based on clinical suspicion can save lives.
• Intraoperative identification of malrotation aids in optimal surgical management.

Clinical timeline: Left-sided perforated appendicitis in a 22-year-female with intestinal malrotation: A case report from a resource-limited setting

**Table:** 

Day	Event
Day 1	Onset of periumbilical abdominal pain
Day 2–3	Pain migrated to the left lower quadrant, anorexia, low-grade fever and nausea
Day 4	worsening abdominal discomfort
Day 5 AM	Abdominal distension observed; presented to emergency department
Day 5 PM	Clinical exam, labs, ultrasound → emergency laparotomy performed
Day 5	Perforated appendix discovered on the left due to intestinal malrotation
Day 6–10	Postoperative recovery (stable clinical course)
Day 11	Discharged home on postoperative day 5

## Patient perspective

While advanced imaging tools were not available for the diagnostic process, the patient expressed gratitude for the prompt diagnosis and surgical treatment she received. She was relieved to learn the reason for her symptoms and satisfied with the care she received throughout the hospitalization and recovery process.

## 1 Introduction

Acute appendicitis is one of the most common surgical conditions requiring immediate intervention. It accounts for approximately 6 % of all emergency department visits. Situs inversus is a rare condition in which asymmetric organs are positioned in mirror images of normal anatomy. The condition may be partial affecting only either the abdominal or thoracic cavities or complete situs inversus totalis: both abdomen and thoracic organs are transposed [1]. Typically, acute appendicitis can be diagnosed by clinical symptoms, physical examination and radiology. However, if the appendix is malpositioned or has anatomical variations, the diagnosis is uncertain and can delay surgical treatment, which can result in complications such as abscesses, perforations, and peritonitis [2]. It is rare for appendicitis to affect the left lower quadrant due to congenital anomalies such as a genuine left-sided appendix or a right-sided appendix that extends into the left lower quadrant [3]. Diagnosis of these cases can be confirmed with imaging revealing an inflamed appendix. However, an abdominal pain in the lower left quadrant may be atypical [4]. This report presents a rare case of left-sided perforated appendicitis due to intestinal malrotation in a 22-year-old female, managed without the aid of CT imaging, because in a resource-limited setting, where early diagnosis was difficult due to the absence of imaging modalities such as CT scan. A completed SCARE checklist is submitted as a supplementary file alongside this manuscript [5].

## 2 Case presentation

During the course of five days, a 22-year-old female presented to the emergency department with periumbilical pain that migrated to the left lower quadrant. She reported nausea, anorexia, low-grade fever, and abdominal distension on the fifth day. Her last menstrual period was one week before admission. She was febrile, not pale or jaundiced, with BP 110/70 mmHg, pulse 100 bpm, RR 20 cpm, and mild distension in the left lower quadrant, rebound tenderness, and a positive left Psoas sign on examination. Organomegally was not observed, and laboratory tests revealed:

• White Blood Count: 42,200 cells/mm^3^ (Neutrophils 86 %)
• Platelets: 305,000/mm^3^
• C-Reactive Protein: 279.9 mg/L
• Potassium: 3.0 mmol/L (hypokalemia)
• Total bilirubin: 2.5 mg/dL (indirect predominant)
• Negative pregnancy test
• Normal renal function
• Urinalysis: pus cells

**Fig. 1:**
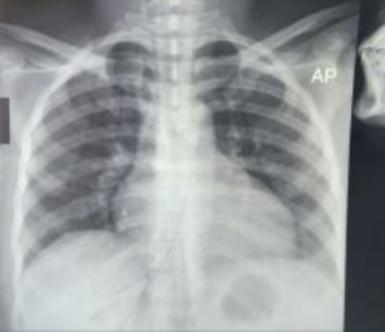
Normal erect chest X-ray excluding free subdiaphragmatic air.

Ultrasound showed distended bowel loops with a non-visualized appendix with pelvic fluid collection. Chest X-ray was normal. (Fig. 1).

**Fig. 2:**
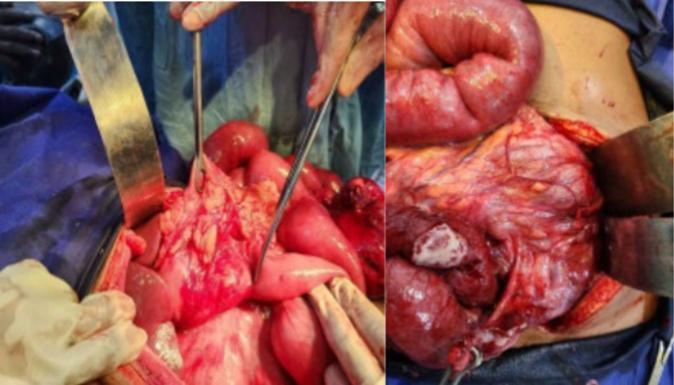
Intraoperative photograph showing inflamed and perforated appendix (arrow) (right) with adjacent cecum, and cecum (left).

**Fig. 3:**
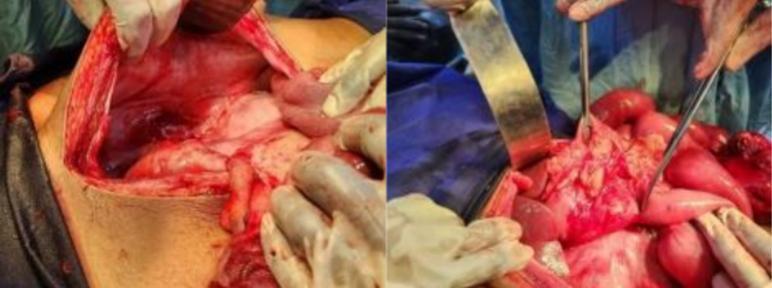
Right empty paracolic gutter (arrow) (left) and DJ flexure positioned abnormally to the right of midline (right).

A provisional diagnosis of generalized peritonitis due to a perforated hollow viscus was made. Emergency laparotomy via midline incision revealed (Fig. 2, Fig. 3):

• 600 mL pus in the left paracolic gutter and pelvis
• Small bowel adhesions to the anterior and left abdominal wall
• Small bowel on the right side, cecum, appendix (perforated at distal third), and ascending, transverse, descending and sigmoid colon on the left
• Greater omentum in the left iliac fossa
• Ligament of Treitz and DJ flexure right of the midline
• Normal liver, spleen, gallbladder, uterus, ovaries, kidneys

Appendectomy was performed via a midline laparotomy incision; a pelvic drain was placed, and the peritoneal cavity was thoroughly irrigated. The patient was managed with broad-spectrum antibiotics (ceftriaxone and metronidazole), potassium correction, and early mobilization. Histopathology confirmed acute appendicitis without evidence of malignancy. The patient recovered well and was discharged on the fifth postoperative day and remained well on follow-up at two weeks and one month.

## 3 Method

This case report has been reported in line with the SCARE 2025 criteria. A completed SCARE checklist is submitted as a supplementary file alongside this manuscript.

This case has been registered with the Research Registry under the Unique Identifying Number (UIN): researchregistry11417.

## 4 Discussion

Approximately 90 % of reported cases of left-sided appendicitis are caused by congenital anatomical variations, such as situs inversus and intestinal malrotation. These conditions alter typical symptom patterns, making diagnosis difficult [6].

Approximately 1 in 10,000 to 50,000 people have situs inversus totalis, a condition in which all thoracic and abdominal organs are inverted, complicating the evaluation of common abdominal conditions [7].

Clinical presentation such as fever or nausea may be mild or absent in cases of left lower quadrant pain and rebound tenderness, resulting from diverticulitis or epiploic appendagitis, not appendicitis [8].

An intestinal malrotation occurs when the midgut cannot rotate around the superior mesenteric artery in the peritoneal cavity and has a fixation problem. According to the literature, midgut malrotation occurs in between 0.03 and 0.5 % of live births, and most cases are diagnosed early in life. Less than 0.5 % of cases are diagnosed in adulthood [9].

Because of significant variations in the location of the vermiform appendix, approximately a third of acute appendicitis patients experience abdominal pain elsewhere than the right iliac fossa. However, acute appendicitis usually does not manifest with left lower quadrant abdominal pain [8]. .In young women, the differential diagnosis of left iliac fossa pain also includes gynecological causes such as ruptured ovarian cyst, ovarian torsion, and pelvic inflammatory disease, as well as gastrointestinal causes like sigmoid diverticulitis and epiploic appendagitis [8 & 9].

The very high leukocytosis and markedly raised CRP in this patient indicated a strong systemic inflammatory response. The associated indirect hyperbilirubinemia further suggested complicated appendicitis with perforation. These laboratory abnormalities are recognized markers of advanced disease, aligning with the intraoperative finding of a perforated appendix. Similar correlations between inflammatory markers and complicated appendicitis have been described in the literature (9 &11).

The use of imaging, particularly CT, aids diagnosis, but isn't available in low-resource settings. Ultrasound is operator-dependent and limited by gas-filled bowel loops [10]. In this patient, the limitation of ultrasound necessitated reliance on clinical judgment and the decision for emergent laparotomy.

Laparoscopic or open surgery is the mainstay of management to avoid complications. Early exploration is justified when imaging is inconclusive and clinical suspicion is high [11].

## 5 Conclusion

Patients with left-sided abdominal pain should be evaluated with a high index of suspicion, especially in resource-limited settings due to non-typical presentations of appendicitis such as intestinal malrotation. In the absence of advanced imaging, clinical suspicion, timely decision-making, and surgical exploration are essential.

## Author contribution

Dr. Abdulrahman Mohammed Abdulrahman Abouh: Conceptualization, surgery, manuscript writing, submission.

Dr. Ahmed Idris: Clinical management, data acquisition, manuscript editing.

Dr. Arwa Mohammed Abdallah Gomaa: Literature review, patient follow-up, formatting.

Prof. Hussain G. Ahmed: Supervision, critical revision, final approval.

## Consent

A written informed consent was obtained from the patient for publication of this case report. A copy of the written consent is available for review by the Editor-in Chief of this journal upon request.

## Ethical approval

In accordance with our hospital's policy, case reports do not require approval, provided a written informed consent has been obtained from the patient.

## Guarantor

Abdulrahman Mohammed Abdulrahman Abouh

## Source of funding

This case report did not receive any form of funding or grant from public, private or non-profit organizations.

## Declaration of competing interest

The authors declare no conflicts of interest regarding the preparation or publication of this case report.
